# Evidence of Long-Lived Founder Virus in Mother-to-Child HIV Transmission

**DOI:** 10.1371/journal.pone.0120389

**Published:** 2015-03-20

**Authors:** Sivapragashini Danaviah, Tulio de Oliveira, Ruth Bland, Johannes Viljoen, Sureshnee Pillay, Edouard Tuaillon, Philippe Van de Perre, Marie-Louise Newell

**Affiliations:** 1 Africa Centre for Health and Population Studies, University of KwaZulu-Natal, Durban, South Africa; 2 Université Montpellier 1, 34090, Montpellier, France; 3 Royal Hospital for Sick Children, Glasgow, United Kingdom; 4 Centre Hospitalier Universitaire de Montpellier, Département de Bactériologie-Virologie, Institut de Recherche en Biothérapie and Department of Medical Information, 34295, Montpellier, France; 5 Faculty of Medicine, University of Southampton, Southampton, United Kingdom; University of British Columbia, CANADA

## Abstract

Exposure of the infant’s gut to cell-associated and cell-free HIV-1 trafficking in breast milk (BM) remains a primary cause of mother-to-child transmission (MTCT). The mammary gland represents a unique environment for HIV-1 replication and host-virus interplay. We aimed to explore the origin of the virus transmitted during breastfeeding, and the link with quasi-species found in acellular and cellular fractions of breast-milk (BM) and in maternal plasma. The C2–V5 region of the env gene was amplified, cloned and sequenced from the RNA and DNA of BM, the RNA from the mother’s plasma (PLA) and the DNA from infant’s dried blood spot (DBS) in 11 post-natal mother-infant pairs. Sequences were assembled in Geneious, aligned in ClustalX, manually edited in SeAL and phylogenetic reconstruction was undertaken in PhyML and MrBayes. We estimated the timing of transmission (ETT) and reconstructed the time for the most recent common ancestor (TMRCA) of the infant in BEAST. Transmission of single quasi-species was observed in 9 of 11 cases. Phylogenetic analysis illustrated a BM transmission event by cell-free virus in 4 cases, and by cell-associated virus in 2 cases but could not be identified in the remaining 5 cases. Molecular clock estimates, of the infant ETT and TMRCA, corresponded well with the timing of transmission estimated by sequential infant DNA PCR in 10 of 11 children. The TMRCA of BM variants were estimated to emerge during gestation in 8 cases. We hypothesize that in the remaining cases, the breast was seeded with a long-lived lineage latently infecting resting T-cells. Our analysis illustrated the role of DNA and RNA virus in MTCT. We postulate that DNA archived viruses stem from latently infected quiescent T-cells within breast tissue and MTCT can be expected to continue, albeit at low levels, should interventions not effectively target these cells.

## Introduction

HIV-1 transmission via breastfeeding accounts for approximately one third to one half of all mother-to-child transmissions (MTCT) in settings where antiretroviral therapy (ART) is not available and breastfeeding occurs for prolonged periods of time[1]. Infected breast milk (BM) contains both cell-free HIV-1 RNA in lactoserum, and intracellular HIV-1 RNA and DNA [2,3]. We have previously shown that cumulative exposure to BM HIV-1 RNA, as well as levels of virus, both RNA and DNA, are major risk factors for postnatal HIV-1 transmission [4,5]. However, antiretroviral treatment (ART) has limited or no effect on the size of cell-associated HIV-1 RNA and DNA reservoir in BM [2,6]. Although both cell-free (RNA) and cell-associated (DNA) virus may be transmitted to the infant, the relative contribution of each to the risk of transmission [7], the timing of transmission[8] and the extent of viral shedding over time [5], have yielded conflicting reports.

A link between the type of HIV-1 reservoir in BM, and the estimated timing of transmission (ETT) to the infant, has been reported. Early postnatal transmission appears to be mainly associated with archived cell-associated virus (HIV DNA), whereas cell-free HIV-1 RNA is more commonly associated with transmission at a later stage [5,8,9]. The mammary gland may be seen as an effector lymphoid organ with strong linkage to the mucosal associated lymphoid tissue (MALT) where continuous cellular exchanges (T- and B-lymphocytes) with mucosal inductor sites occur [2]. Breast milk lymphocytes are highly activated and have a phenotype that differs notably from their blood counterparts [3]. This, and data from previous studies suggest that the mammary gland could constitute a proper compartment of HIV-1 replication [9,10,11]. Activation of latently infected immune cells occurring during the migration and homing of CD4+ T-cells to the mammary gland may be conducive to divergent viral evolution. However, other reports suggest that BM does not represent a site of divergent viral evolution, but contains a dynamic intermixing of plasma- and breast milk-derived variants with multiple infecting lineages [11]. There is a need to further explore this viral genetic bottleneck between mother and infant during breast-feeding transmission of HIV-1.

## Methods

### Ethics statement

The Biomedical Research Ethics Committee (BREC) of the University of KwaZulu-Natal approved the Vertical Transmission Study (VTS) and this analysis (Reference number T050/01). All study participants provided informed, written consent following administration of a Consent form in both English and Zulu, the local vernacular language. Written consent was provided for maternal and infant sample collection, storage as well as for all subsequent molecular analyses such as we describe for our study.

### Study Design and Sample Collection

This study was nested within the Vertical Transmission Study (VTS) conducted between August 2001 and September 2006 in the uMkhanyakude District of KwaZulu-Natal, South Africa [12,13]. This non-randomized intervention cohort study investigated the impact of breastfeeding practices on postnatal HIV-1 transmission, infant mortality and morbidity in a rural setting. Single‐dose nevirapine (sdNVP) was administered to all HIV-1 infected mothers during labor/delivery, and to their newborns as per the national guidelines at the time [14]. All mothers received counselling and support regarding breastfeeding practices. None of the breast milk samples used in this analysis were collected from mothers who had reported breast health problems at the time of sampling, but one mother (Case 21) reported breast health problems over the duration of breastfeeding. Maternal plasma (PLA) and BM samples, as well as infant dried blood spots (DBS) were collected at scheduled clinic visits and transported to the Africa Centre Virology Laboratory for processing.

**Fig 1:**
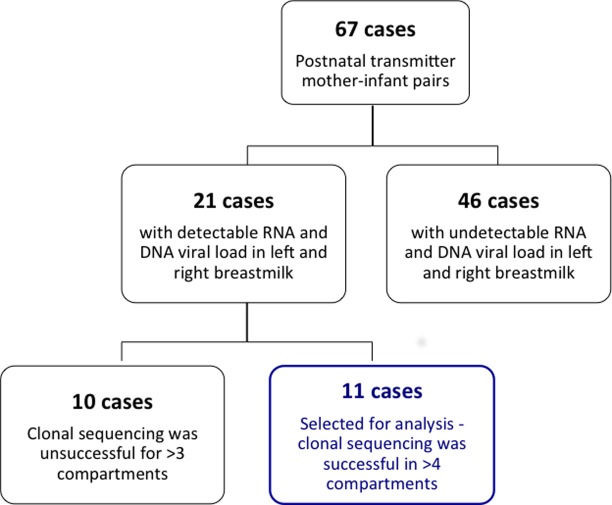
Sample size of VTS cohort. An illustration of the number of patients in the post-natal vertical transmission study and the subsequent number of cases, comprising mother-infant pairs, selected for this analysis.

A total of 67 infants were identified as having acquired HIV-1 via breast milk following a negative PCR test at 6-weeks of age but a subsequent positive PCR result. The majority of these post-natal transmitter pairs (Fig. 1) could not be analysed given that there was undetectable viral load in at least one of the four breast milk compartments (RNA, DNA, right and left BM). The remaining twenty-one pairs with proven postnatal transmission of HIV-1 were selected for this analysis on the basis of having detectable HIV-1 RNA and DNA viral loads in both left (LBM) and right BM (RBM) samples at 6 weeks and 6 months post-delivery (Fig. 1). We chose to analyse left and right breast milk samples independently for two reasons: firstly we noted significantly disparate HIV RNA and DNA viral loads between breasts [5,15]; and secondly to account for the effect of “dominant-breast” feeding practices and the related physiological effect of engorgment and accumulation of viral variants. The breast milk samples chosen for this analysis did not coincide with clinical symptoms or reports of breast health problems at the time of sample collection. Infants were all negative for HIV-1 RNA from DBS taken at 6 weeks of age but tested positive subsequently (Nuclisens EasyQ HIV-1 assay, Biomerieux, Boxtel, Netherlands). The estimated timing of transmission (ETT) was defined as the mid-point between the infant’s last negative and first positive HIV-1 test as defined in a previous report on the VTS study cohort [13]. The BM sample chosen for sequence analysis was one collected at a time point prior to the ETT in order to trace the virus that was transmitted to the infant. Similarly, the maternal plasma sample chosen for analysis was one that was collected at a time point closest to the ETT (either the 6 week (n = 16) or 6 month (n = 5) postnatal sample). The first HIV-1 positive infant DBS was selected for clonal sequencing. For one case (Case1) we chose to analyse the 6 week and the 6 month breast milk sample in order to investigate whether there were temporal differences in viral evolution.

### Nucleic Acid Extraction

HIV-1 RNA was purified from 140μl maternal plasma (stored at -80°C) using the QIAamp Viral RNA Mini Kit (Qiagen). A single DBS spot (50μl whole blood) was used for HIV-1 DNA extraction (QIAamp Blood DNA Mini Kit, Qiagen). DBS was not available for 3 infants therefore we extracted RNA from available plasma and proceeded as for the maternal plasma RNA. Whole BM, stored at -80°C, was thawed and centrifuged (1200g, 15minutes) to separate breast milk cells from lacto-serum. BM HIV-1 RNA and DNA were extracted from 500μl of lactoserum (Abbott ASPS magnetic extraction system, Abbott), and cell pellets (QIAamp Blood DNA Mini Kit, Qiagen), respectively. Breast milk RNA extracted products were not DNase treated prior to amplification therefore may include a minor DNA component. Given that thawing the whole breast milk may have resulted in cell rupture, we acknowledge that there may have been limited mixing of RNA and DNA in the lactoserum and cell pellet.

### HIV-1 Viral Load Quantification

HIV-1 RNA viral load was quantified from maternal plasma and infant DBS as described previously [13]. BM HIV-1 RNA viral load (copies/ml) was quantified from 500μl BM lactoserum using the Biocentric Generic HIV Charge Virale kit (Biocentric, Bandol, France) with a lower limit of detection of 375cp/ml of lactoserum, and HIV proviral DNA load (copies/10^6^ BM cells) was quantified using the Biocentric DNA Cell assay (Biocentric, Bandol, France) with a lower limit of detection of 1cp/10^6^ BM cells, as previously described[4,5].

### Amplification of *env* Gene, Cloning and Sequencing

HIV-1 RNA was reverse transcribed (20μl final volume) using SuperScript III Reverse Transcriptase (Invitrogen, Life Technologies, Foster City, USA). The C2–V5 region of the HIV-1 *env* gene (621bp, nucleotides 7026–7647, HXB2 numbering) was PCR amplified from the reverse transcribed RNA of the mother’s plasma, left and right breast milk lactoserum and the DNA of the mother’s left and right breast milk sample as well as the infant’s DBS with primers MK605 (5'-AATGTCAGCACAGTACAATGTACAC-3'; positions 6945 to 6969) and CD4R2 (5'-TATAATTCACTTGTCCAATTGTCC-3') as outer and M13F-ES7 (5’-tgtaaaacgacggccagtCTGTTAAATGGCAGTCTAGC-3’; position 7002–7021) and M13R-ES8 (5’-caggaaacagctatgaccCACTTCTCCAATTGTCCCTCA-3’; positions 7648–7668) as inner primers, as previously described[16]. In this way, both the cell-associated (breast milk DNA) and cell-free viral populations within the breast milk were amplified. Positive PCR products were purified (PureLink PCR Purification kit, Invitrogen) and cloned into the pCR4-TOPO vector of the TOPO TA Cloning Kit for Sequencing (Invitrogen). Transformed cells were screened for uptake of the insert (40ng/μl X-gal) and positive clones were purified (Promega) and confirmed by restriction digest (EcoR1) and PCR amplification. Bi-directional sequencing was performed on 10–20 positive clones per compartment with universal T3 (5’-ATTAACCCTCACTAAAGGGA-3’) and T7 (5’-TAATACGACTCACTATAGGG-3’) primers on an ABI 3130xl Genetic Analyzer (Life Technologies).

### Sequence Editing, Alignment and Phylogenetic Analysis

Chromatograms were imported and visually edited in Geneious V6.1.8 [17]. Sequences with quality scores exceeding 90% were accepted for analysis. Amplification of *env* gene and cloning and sequencing yielded sequence data in 11 out of 21 postnatal transmitter pairs selected for analysis. Ten cases were excluded because sequence data could not be produced for >3 of the 6 compartments (8/10 cases) or we noted evidence of non-C subtypes (2/10 cases) suggesting inter-laboratory contamination during the cloning procedure. We assessed contamination in two ways, as we have in previous publications[18,19]; firstly by phylogenetic reconstruction of sequences generated in this analysis with all other HIV sequences generated at our facility and secondly, by determining the sequence subtype of all clonal sequences using the Rega Subtyping tool V3[20]. In this way we confirmed that sequences chosen from the 11 pairs for further analysis were free of inter-patient or inter-laboratory contamination. Consensus sequences (~621bp) were generated in Geneious, aligned using ClustalX[21] and manually codon-edited in Se-AL (http://tree.bio.ed.ac.uk) (codon alignments available on request). Phylogenetic reconstruction of maximum likelihood (ML) trees was performed in PhyML [22] and Bayesian trees in MrBayes[23]. The trees were constructed using models estimated with Akaike selection in ModelTest [24]. Gamma heterogeneity alpha parameter was estimated in the ML and Bayesian tree construction process. The ML tree topology was optimized using SPRs method and a BioNJ starting tree in PhyML. The reliability of the internal nodes was estimated using 100 Bootstrap replicates (ML trees) and 4MCMC chains with 10^6^ generations sampled every 10^3^ generations and a 10% burning (Bayesian trees). Results were visualized using Tracer where parameters with an effective sample size (ess) >150, suggesting good mixing and sampling of the trees, were accepted. All consensus trees were generated in TreeAnnotator with posterior support for internal branches estimated. To further investigate the pathway of transmission and to estimate the most recent common ancestor (TMRCA) of each dataset, sequences were analyzed in BEAST1.6 (http://beast.bio.ed.ac.uk) [25]. A simple, constant population size coalescent model was used to generate an initial tree followed by a general time reversible (GTR) substitution model (gamma distribution). To estimate the time of divergence of each compartment, we applied an uncorrelated relaxed clock, gamma distribution [23], an initial chain of 10^8^ and 10% burning. Results were accepted if effective sample size was >150. We investigated the impact of time on viral migration and shifts in viral populations by analyzing data generated from the two time points sequenced for Case1. All analyses as described above was applied to the complete dataset and illustrated in Phylotype (www.phylotype.org)[26]. Sequences and their annotations (compartment and time point) were analyzed with the following criteria; size (5), persistence (1), size/difference in population size (1) and support (0.75) with 500 iterations. Highlighter plots were constructed for each dataset against a consensus sequence of the breast milk and another of the plasma sequences for each case (http://www.hiv.lanl.gov/content/sequence/HIGHLIGHT/highlighter_top.html) in order to identify the founder virus in each compartment[27]. All sequences are publically available from Genbank (Accession numbers KP261088-KP261820).

### Statistical Analysis

Data were analyzed using Prism v5 and SPSS v19. Given the small sample size, non-parametric analyzes were conducted and included univariate analyzes of variance (One-way ANOVA) and tests of correlation (Spearman’s Rank test and Kendall’s test). Statistical significance was accepted at a 1% or 5% level of significance.

## Results

Viral load measurements in the infant DBS and mother’s plasma samples were significantly greater (p<0.05) than all breast milk viral load measurements (DNA and RNA, Table 1) but were comparable to each other. We chose to investigate the left and right breast independently given the variation in viral load between the breasts, which we hypothesized would result in differential evolutionary kinetics. As such we had 6 compartments comprising BM defined as left and right BM RNA and DNA, maternal PLA RNA and infant DBS DNA.

**Table 1:** A summary of the clinical data of the 11 mother-infant pairs selected for clonal sequence analysis.

Case number	Breast milk (BM) viral load				Infant’s DBS viral load	Mother’s plasma (PLA) viral load
	LBM-DNA	RBM-DNA	LBM-RNA	RBM-RNA	(log cp/ml)	(log cp/ml)
	(log cp/10^6^ cells)	(log cp/10^6^ cells)	(log cp/ml)	(log cp/ml)		
1	4.59	4.75	5.06	5.26	4.49	ND
3	5.14	4.35	3.95	3.92	5.85	5.53
4	4.61	4.27	4.30	3.91	4.38	4.97
10	4.52	4.65	4.52	3.85	4.56	5.75
11	3.68	3.87	3.74	3.66	5.63	5.68
12	3.97	<LDL	3.37	3.58	6.12	5.61
14	4.30	4.48	4.23	4.32	4.86	5.42
15	4.36	5.72	4.68	5.60	4.49	4.76
18	4.07	4.50	3.19	4.03	3.64	5.43
19	3.76	4.16	3.73	3.86	6.72	6.10
21	4.89	4.60	5.36	4.10	5.81	6.78
**Mean ± SD (range)**	**4.35±0.46**	**4.19±0.68**	**4.53±0.46**	**4.19±0.65**	**5.14±0.93**	**5.60±0.56**
	**(3.68–5.14)**	**(3.19–5.36)**	**(3.68–5.72)**	**(3.58–5.60)**	**(3.64–6.72)**	**(4.76–6.78)**

Key: <LDL = < the lower detectable limit of the test (6cp/10^6^ cells); ND = not determined

From the eleven mother-infant pairs we produced 733 sequences from the 6 sources of viral genetic material (mean number of sequences per source = 122; range = 109–127). The lowest level of intra-compartment genetic distance was observed for infant DBS samples (Table 2), with significantly lower quasi-species diversity than in maternal samples (PLA, BM DNA and RNA) (Table 2). In contrast, the infant datasets displayed greater sequence homology, compared with the breast milk and plasma sequences, characteristic of archived virus and recency of infection.

**Table 2:** Mean intra-compartment genetic diversity of the six compartments sequenced, namely Infant blood, Mothers plasma, BM-RNA and BM-DNA, as calculated in MEGA Version 6.06.

CASE No.	Mean intra-compartment genetic distance					
	INFANT-DBS (n = 11)	MOTHER’S PLA-RNA (n = 11)	LBM-DNA (n = 10)	LBM-RNA (n = 10)	RBM-DNA (n = 10)	RBM-RNA (n = 10)
1	0.035	0.061	0.022	0.039	0.000	0.081
3	0.035	0.019	0.018	0.022	0.020	0.050
4	0.013	0.035	0.032	0.002	0.039	0.001
10	0.002	0.050	0.020	0.021	0.026	0.014
11	0.005	0.005	ND	0.004	ND	0.003
12	0.004	0.012	0.026	0.001	0.016	ND
14	0.001	0.024	0.026	0.011	0.012	0.014
15	0.003	0.016	0.044	0.046	0.028	0.051
18	0.001	0.014	0.001	ND	0.001	0.014
19	0.001	0.000	0.003	0.018	0.009	0.011
21	0.040	0.055	0.043	0.036	0.016	0.027
Mean diversity ± SD (Range)	0.013±0.016 (0.001–0.040)	0.026±0.021 (0.000–0.061)	0.024±0.014 (0.010–0.044)	0.020±0.016 (0.001–0.046)	0.017±0.021 (0.000–0.039)	0.027±0.026 (0.001–0.081)

Key: ND = not determined as clones from these compartments could not be generated, LBM-DNA = left breast milk DNA, LBM-RNA = left breast milk RNA, RBM-DNA = right breast milk DNA, RBM-RNA = right breast milk RNA, 1 = T-test p = 0.01, 2 = T-test p = 0.04.

### The Number and Origin of Transmitted HIV Strains

**Fig 2:**
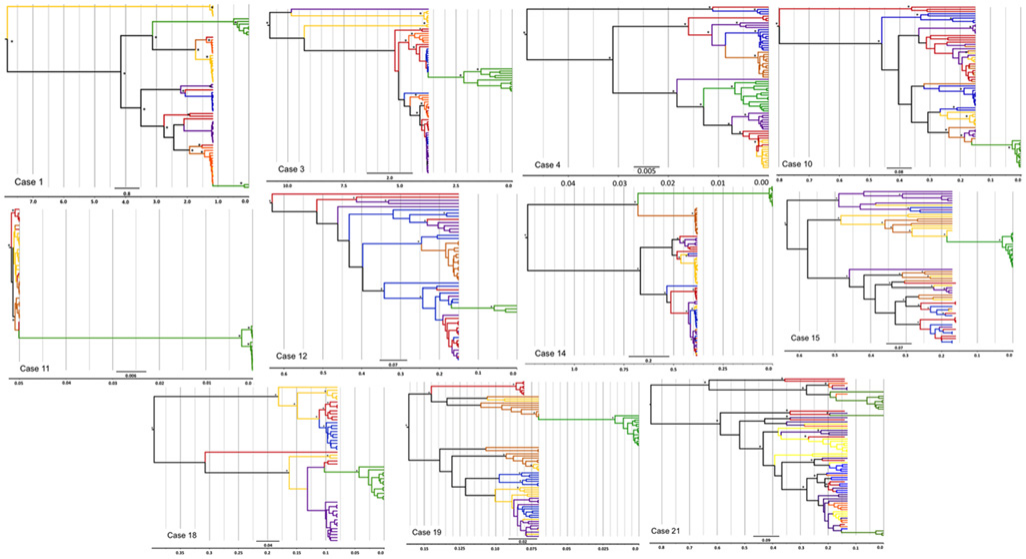
Phylogenetic reconstruction of the most recent common ancestor (TMRCA). This image illustrates the Bayesian maximum clade credibility (mcc) trees inferred in BEAST. Terminal branch colors represent sequences derived from the infant DBS DNA (green), maternal plasma (red), left breast milk DNA (purple), left breast milk RNA (orange), right breast milk DNA (blue) and right breast milk RNA (yellow). Unambiguous internal branches are shaded according to the compartment of origin or shaded black if the origin was ambiguous. Timelines indicate the number of years in the past from the date of sample collection of the infant DBS, indicated as Time 0.

Phylogenetic reconstruction illustrated the direct ancestry of infant HIV-1 quasi-species to maternal viral variants. Distinct monophyletic clusters of infant sequences were observed where a single variant was acquired and expanded to form a distinct phylogenetic branch. In 9 of 11 cases, the infant acquired one major strain during breastfeeding (Fig. 2), which then expanded within the infant. There was evidence of acquisition of multiple variants in Cases 1 and 21; with two and four HIV-1 variants of maternal origin detected within the infants DBS sequence clusters.

Sequences were analyzed to determine whether viral transmission most likely involved a cell-associated DNA or cell-free RNA source, based on sequence homology (Fig. 2). In four cases, the virus infecting the infant most likely originated from the cell-free RNA (Cases 1, 14, 15 and 19), whereas cell-associated DNA was implicated in two cases (Cases 4 and 12). It was not possible to specify an appropriate cell-free RNA or cell-associated DNA source in the remaining five cases. Using phylogenetic analysis we identified the source of transmitted virus i.e. which breast was involved (left/right). We noted discrete breast milk-derived sequence clusters in 5 of 11 cases. We identified the mammary gland and viral source most probably involved in transmission in these cases with 4 cases involving the left breast (Cases 4, 10, 14, 19), and 1 the right breast (Case 15). The monophyletic clustering of sequences corresponding to anatomical compartments was further tested using nucleotide-by-nucleotide sequence comparison (Highlighter plots, http://www.hiv.lanl.gov/content/sequence/HIGHLIGHT/). Variants with complete homology to the breast milk consensus sequence were noted within the infant compartments in 7 of 11 cases (S1). Sequences homologous to the mother’s plasma were noted among breast milk RNA and DNA quasi-species. We thereby demonstrate the presence of breast milk founder virus within the infant dataset and plasma founder variants within the breast milk dataset.

### Molecular Clock Analysis, Timing of Transmission

**Fig 3:**
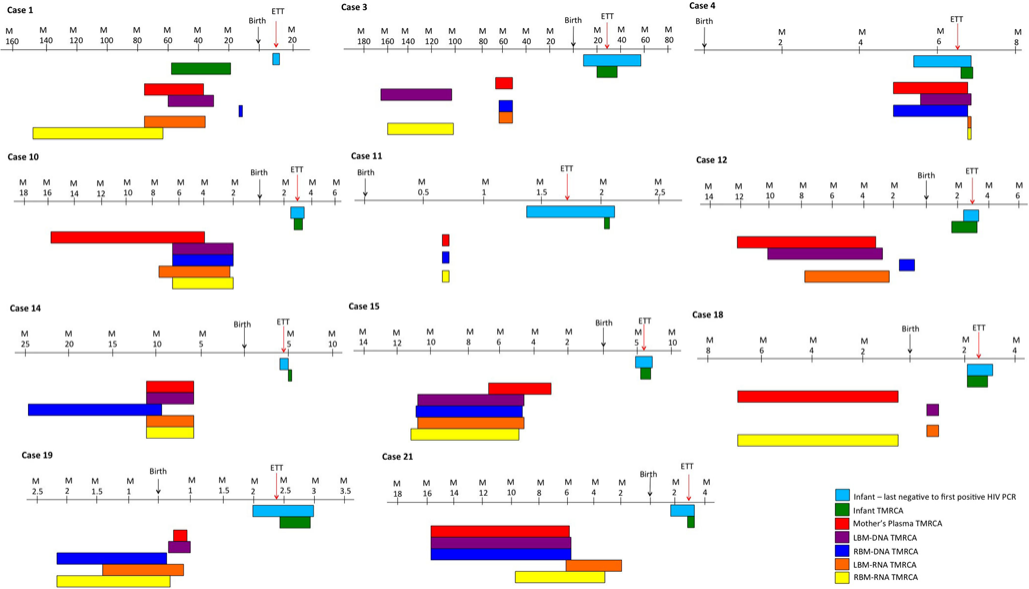
Comparison of TMRCA estimates. Graphical representations of the upper and lower limit (95% CI) of the most recent common ancestor (TMRCA) estimations of each compartment of the datasets as calculated in BEAST for the infant DBS DNA (green), the mother’s plasma RNA (gray), the left breast milk DNA (purple), left breast milk RNA (yellow), right breast milk DNA (red) and right breast milk RNA (blue).

Phylogenetic analysis was used to determine the ETT of virus that the infant based on diversity growing (Fig. 3) and compared it to the reference ETT based on sequential molecular testing. We noted a median ETT based on phylodynamics of 12 ±4.8wks (IQR = 14wks) match with the reference ETT in all the cases (Fig. 2). The median time to the most recent common ancestor (TMRCA) estimated from sequence data for the infants was 10±18wks (IQR = 13wks) with an overall mean difference between these estimates of 6.6wks. The TMRCA was closely associated with the ETT where the time in weeks between the date of delivery and the TMRCA compared with the ETT was not significantly different (p>0.05) in all but one case. In Case 1 (Fig. 3, Case1), the ETT was approximately 6 months after birth, whereas the Bayesian molecular clock analysis placed the infant’s TMRCA during gestation, an indicator of a possible ancient or archieved viral lineage.

The TMRCA of HIV-1 DNA and RNA in the mother’s BM was also calculated, using the same approach. The mean TMRCA of the DNA and RNA compartments was estimated to fall within the period of gestation in eight cases. This suggests that HIV-1 was introduced in the breast during pregnancy. In two remaining cases (Case 1 and 3), BM TMRCA dated to several years prior to the infant’s date of birth suggesting that old archived strains were introduced to the breast. In the remaining case the TMRCA of the BM dataset (RNA and DNA) were dated to within days of the infant’s TMRCA (Fig. 2, Case 4).

## Discussion

Although breast milk accounts for tens of thousands of new HIV-1 infections each year, there are few data on the origin and routes of transmission of HIV-1 variants via breast milk. Using clonal sequencing and advanced computational tools, we investigated the genetic complexity of HIV-1 in the mother-offspring dyad. Our data evidenced the formation of a genetic bottleneck during postnatal HIV-1 transmission from mother-to-child with panmictic breast milk viral populations. In a recent publication, Chaillon et al [28] used the same analytical approaches to study the transmitted virus in mother-infant pairs using longitudinal blood samples. Although breastfed infants were exposed several times daily to HIV-1 infected cells (HIV DNA) and cell-free particles (HIV RNA), analysis of the complexity of the transmitted virus among breastfed infants confirmed that transmission is predominantly via a single variant [10,11,29]. The rate of multiple variant transmissions was not higher than that observed for heterosexual HIV-1 transmissions or intravenous drug users [27,30,31] and is likely to have occurred over time and on multiple occasions. Conversely, a predominant variant may have been transmitted multiple times over the course of breastfeeding but our analysis did not attempt to resolve this dynamic. Our findings are in line with previous reports investigating early and late HIV transmission from a mother to her infant [32,33].

The monophyletic clustering patterns clearly differentiating left and right, RNA and DNA viral quasi-species from our data had high branch support. We hypothesize that the difference in clustering patterns reflects the kinetics and clustering of quasi-species between productive viremia (RNA) versus archived virus populations (DNA). These patterns are also influenced by the degree of engorgement due to limited feeding and thus viral clearance from each breast. We have, with this cohort, demonstrated the impact of long-term exposure to RNA versus DNA virus and the risk of transmission in a previous publication [15]. In addition, we noted that parity seems to influence the complexity of the transmission pattern and viral kinetics but given that this is a small cohort (11 cases) with limited data points, we are unable to test this hypothesis with confidence.

The level of maturation of the infant’s gut, mixed feeding and inflammation of both mammary gland and infant gut may all impact on the risk of transmission and the nature of the transmitted virus. The gut-associated lymphoid tissue and intestinal mucosa—a simple columnar epithelium—has an outsized surface area of approximately 200m^2^ and constitutes a vast portal of entry to both cell-free and cell-associated HIV-1. Analysis of sequence homology from BM HIV-1 DNA and RNA was used to determine the various contribution of cell-free and cell-associated virus during transmission. The source of HIV-1 strain infected the child was determined for approximately half of mother-offspring pairs and involved both cell-associated DNA and cell-free RNA. By analyzing viral variants from the each breast separately, we were also able to demonstrate the possible impact of differential milk output between right and left breasts [34].

In this study we observed that transmission due to cell-associated DNA compared with transmission due to cell-free RNA occurred at comparable time points, whereas previous studies noted earlier transmission was largely due to cell-associated virus while later transmission implicated cell-free RNA resulting from cumulative exposure [4,5,8]. However, it must be noted that our study was of a smaller cohort and our observations would require verification in a larger cohort. Although there is continuous traffic of infected cells and cell-free virus between the mucosal immune system and blood, a certain degree of compartmentalization of HIV does occur in the mammary gland [10,11]. Our study showed that ETT based on growing HIV diversity provides an accurate estimation of the time of HIV transmission. TMRCA in the child, based on HIV genetic diversity, confirmed the ETT based on longitudinal monitoring of detectable viral DNA in 10 of 11 infants in our study. Similarly, Chaillon et al [28] used Bayesian inference of transmission and sequence data to accurately confirm the timing of transmission from mother to child as estimated by longitudinal PCR testing.

Our analyses of breast milk TMRCA further suggested that the growing diversity in the mammary gland started during pregnancy, in some cases several months before lactation. In three cases, the BM TMRCA was estimated to be several years prior to the current pregnancy clearly illustrating the influence and presence of long-lived ancestral lineages within the breast that were established during a previous pregnancy. During pregnancy important tissue changes occur in the mammary gland and immune factors such as lymphocytes home to this tissue[35]. We found that the left mammary gland most likely harbored the transmitted viral variant in four cases compared with one case associated with the right breast. This disequilibrium between mammary glands may reflect a fortuitous finding given our small sample size, however, we cannot exclude that this observation potentially reflects a consequence of breastfeeding practices where one breast is preferentially used most often as a matter of convenience to the mother or as a result of greater breast milk output [34].

As previously noted, the incidence of severe breast health problems was low (<1%) in the VTS cohort from which our study samples were extracted [36]. However, this does not preclude asymptomatic subclinical mastitis, which could influence viral reactivation, expansion and transmission to the infant. Only one mother (Case 21) reported repeated episodes of breast health problems between sample collection time points. She reported bilateral cracked nipples with bleeding on days 25 and 29, and without bleeding on days 54 and 72 after birth. Interestingly, in this case we demonstrated that multiple transmissions of HIV-1 variants to the infant could be traced directly to a maternal plasma variant with TMRCAs between 19–37 days postnatal, suggesting a direct link to the pathologies noted on days 25 and 29.

We demonstrated sequence homogeneity of quasi-species within compartments at the single nucleotide level (S1). The patterns of homogeneity with either the breast milk or plasma consensus sequences indicated the origin of the virus and suggested an expansion of the founder virus within these compartments that originated either from the breast milk in the case of the infants or from plasma in the case of the breast compartment. Mixed variants homologous to the breast milk consensus and the plasma consensus were present in the infant and in the breast compartment reflect the panmictic phenomenon that has been reported previously [10,11,28].

The authors acknowledge the limitations associated with the methodology used to generate the sequence data, that of a population PCR and cloning. Using clonal sequencing to identify minority quasi-species has garnered much criticism. Many advocate single genome amplification in order to exclude the possibility of re-sequencing[10]. However, a comparison of these techniques has found no evidence of re-sequencing, difference in population structure or sequencing bias[37], but large dataset (>10 sequences per compartment) were recommended to eliminate the likelihood of re-sequencing. The use of cross-sectional datasets representing a single time point has been criticized as confounding interpretation of viral population diversity[11]. We nonetheless demonstrated the same pattern of viral migration and heterogeneity as reported in longitudinal datasets[11], indicating that careful laboratory technique and advanced computational analysis could accurately estimate complex transmission patterns even in cross-sectional datasets. This cohort of pregnant women received single dose NVP in order to prevent MCTC, but new guidelines have been implemented since this study was undertaken. Therefore we were unable to test the impact of multi-drug ART regimens on the transmission dynamics and viral kinetics of HIV. A further limitation of the study is that we did not separate immune cells on breast milk collection but stored whole breast milk for later analysis. As such we were unable to investigate the contribution of specific cell types to HIV transmission in MTCT. However, a previous study by Valea et al [3] did illustrate that CD4+ T-cells contribute to productive viremia regardless of ART exposure. These limitations notwithstanding, our study does represent one of the largest breast milk derived clonal sequence datasets from one of the largest, most well-documented postnatal MTCT cohorts analyzed to date.

## Conclusions

The genetic bottleneck that forms during BM HIV-1 transmission appears to be mediated by selection from within the BM in half of our cases. We confirmed that transmission primarily involves one major variant and that the ETT can be appropriately estimated using molecular clock analysis. Using advanced phylogenetic tools we established that the most recent common ancestor of the major HIV variant transmitted to the children by breastfeeding, established in the breast tissue during mammary development, either during the present or a previous pregnancy. Our findings clearly illustrate the complexity of HIV-1 transmission via breast milk from mother to infant and highlight the importance of viral load monitoring during pregnancy and breastfeeding, combination ART and early infant diagnosis and monitoring to prevent ongoing post-natal MTCT. While effective pMTCT programs have reduced transmission rates to <2% in some settings the persistence of breast milk reservoirs will undoubtedly ensure continued, low-level MTCT transmission.

## Supporting Information

S1 Fig Highlighter plots comparing nucleotide sequences generated from the 6 compartments sequenced (BM DNA, BM RNA and mother’s PLA and the infant’s DBS). Each sequence is compared, nucleotide-by-nucleotide, against a consensus of mother’s plasma sequences (red) and a consensus of the breast milk sequences (blue) for each case. Nucleotide positions homologous to the BM consensus are indicated by blue vertical bars and nucleotides homlogous to the plasma consensus are indicated by red vertical bars. Sequences were not sorted according to matches but have, instead, been grouped according to dataset. Homology or matches provide evidence of a founder variant.
(TIF)
