# Supplementary figures and images for: Evidence of Long-Lived Founder Virus in Mother-to-Child HIV Transmission
Source: PLoS One. 2015 Mar 20;10(3):e0120389. doi: 10.1371/journal.pone.0120389 (PMC4368793; doi:10.1371/journal.pone.0120389)

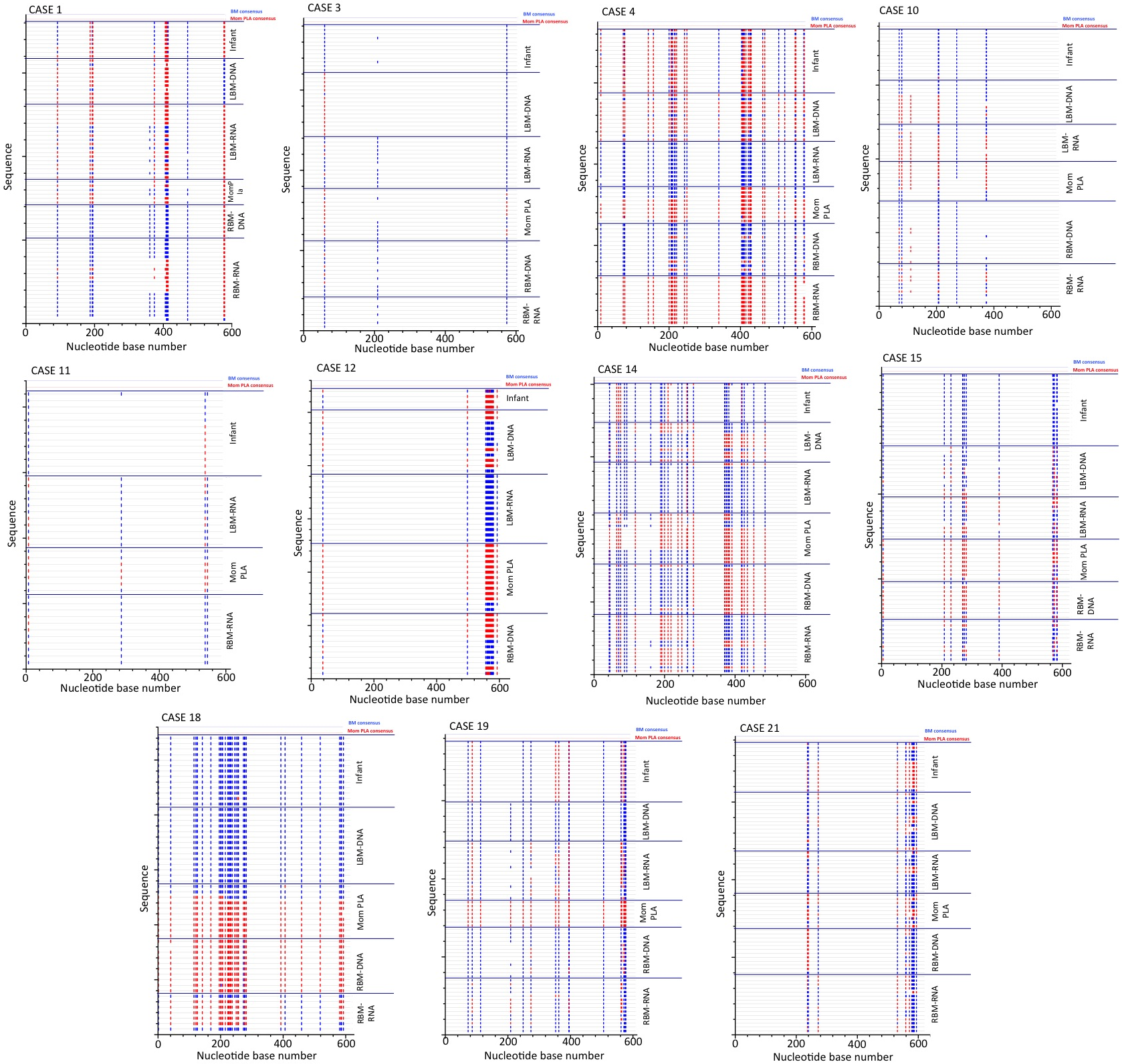

Supplement: S1 Fig — Each sequence is compared, nucleotide-by-nucleotide, against a consensus of mother’s plasma sequences (red) and a consensus of the breast milk sequences (blue) for each case. Nucleotide positions homologous to the BM consensus are indicated by blue vertical bars and nucleotides homlogous to the plasma consensus are indicated by red vertical bars. Sequences were not sorted according to matches but have, instead, been grouped according to dataset. Homology or matches provide evidence of a founder variant. (TIF) [file pone.0120389.s001.tif]
